# Supplementary material for: High-security learning-based optical encryption assisted by disordered metasurface
Source: Nat Commun. 2024 Mar 23;15:2607. doi: 10.1038/s41467-024-46946-w (PMC10960874; doi:10.1038/s41467-024-46946-w)
Supplement: Supplementary file 1 — Supplementary Information [file 41467_2024_46946_MOESM1_ESM.docx]

**Supplementary of “****High-security Learning-based Optical Encryption assisted by Disordered Metasurface”**

**Supplementary Note 1: The derivation of transmission phase and geometric phase**

A simplified Jones matrix $\left[ \begin{matrix} e^{1i*\varphi_{\mathrm{xx}}} & 0 \\ 0 & e^{1i*\varphi_{yy}} \end{matrix} \right]$ can be used to describe the transmission characteristic of the anisotropic meta-pillar. The meta-pillar imposes phase delay ($\varphi_{xx}$ and $\varphi_{yy}$) onto the incident light. $\varphi_{xx}$and $\varphi_{yy}$ symbolize the phase response for the x-x polarization and y-y polarization transmission. The geometric phase can be introduced by rotating the meta-pillar. In this, the Jones matrix used to describe the propagation phase and the geometric phase in the basis of linear polarization can be expressed as

$$\begin{aligned} T_{Linear}=\left[ \begin{matrix} T_{xx}^{f} & T_{xy}^{f} \\ T_{yx}^{f} & T_{yy}^{f} \end{matrix} \right]=\left[ \begin{matrix} \cos\left( \delta\right) & \sin\left( \delta\right) \\ -\sin\left( \delta\right) & \cos\left( \delta\right) \end{matrix} \right]\left[ \begin{matrix} e^{1i*\varphi_{\mathrm{xx}}} & 0 \\ 0 & e^{1i*\varphi_{yy}} \end{matrix} \right]\left[ \begin{matrix} \cos\left( \delta\right) & -\sin\left( \delta\right) \\ \sin\left( \delta\right) & \cos\left( \delta\right) \end{matrix} \right], \#\left( 1 \right) \end{aligned}$$

where $\delta$ is the rotation angle of the meta-pillar. The Jones matrix in the basis of right-handed and left-handed circular polarization can be transformed from that in the basis of linear polarization^1^:

$$\begin{aligned} T_{circular}=\left[ \begin{matrix} T_{LL}^{f} & T_{LR}^{f} \\ T_{RL}^{f} & T_{RR}^{f} \end{matrix} \right]=\frac{1}{2}\left[ \begin{matrix} T_{xx}^{f}+T_{yy}^{f}+i\left( T_{xy}^{f}-T_{yx}^{f} \right) & T_{xx}^{f}-T_{yy}^{f}-i\left( T_{xy}^{f}+T_{yx}^{f} \right) \\ T_{xx}^{f}-T_{yy}^{f}+i\left( T_{xy}^{f}+T_{yx}^{f} \right) & T_{xx}^{f}+T_{yy}^{f}-i\left( T_{xy}^{f}-T_{yx}^{f} \right) \end{matrix} \right] \\ =\frac{1}{2}\left[ \begin{matrix} e^{1i*\varphi_{\mathrm{xx}}}+e^{1i*\varphi_{yy}} & {(e}^{1i*\varphi_{\mathrm{xx}}}-e^{1i*\varphi_{yy}})e^{1i*2\delta} \\ {(e}^{1i*\varphi_{\mathrm{xx}}}-e^{1i*\varphi_{yy}})e^{-1i*2\delta} & e^{1i*\varphi_{\mathrm{xx}}}+e^{1i*\varphi_{yy}} \end{matrix} \right]\#\left( 2 \right) \end{aligned}$$

The phases of $T_{LR}^{f}$ and $T_{RL}^{f}$ are defined by $arg\left( {(e}^{1i*\varphi_{\mathrm{xx}}}-e^{1i*\varphi_{\mathrm{yy}}})/2 \right)\pm2\delta$. The former term of the polynomial is determined by the structure size of the meta-pillar and the latter is dependent on the rotation angle of the meta-pillar, corresponding to the propagation phase and geometric phase, respectively. The phase profiles for two orthogonal circular polarizations (RCP and LCP) are shown in Supplementary Fig. 1. According to Eqs. 2 and 3 in the main text, the required propagation phase and geometric phase can be calculated. In this experiment, the propagation phase (0-2π) is discretized into eight-level phase steps and geometric phase (0-2π) is discretized into 256-level phase steps.


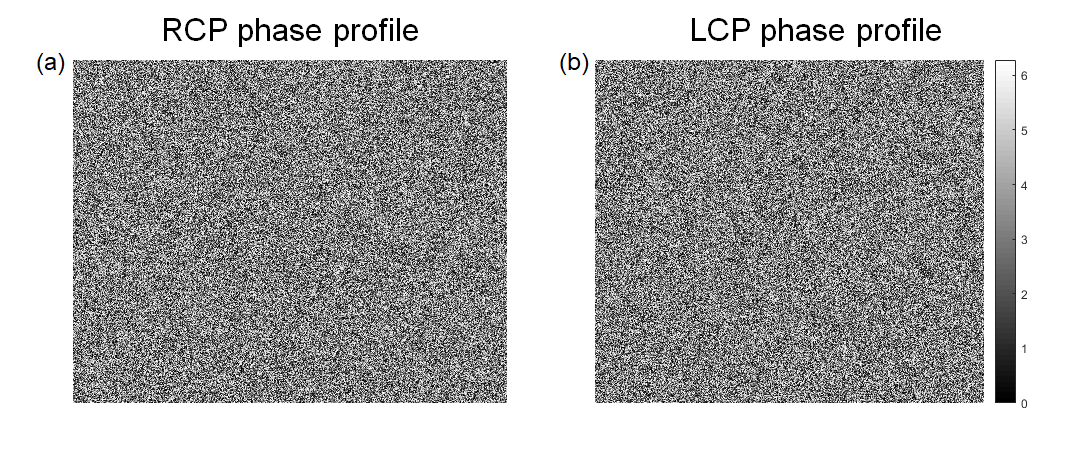


Supplementary Fig. 1 The phase profiles for two orthogonal circular polarizations (RCP and LCP).

**Supplementary Note 2: Parameters of meta-pillars for desired propagation phases**

The structure sizes the propagation phase can be found according to the formula ($\phi_{propagation}=arg\left( {(e}^{1i*\varphi_{xx}}-e^{1i*\varphi_{yy}})/2 \right)$) and Fig. 2b in the main text. The propagation phases are divided into 8-level phases covering 0-2π and the phase value of each level are listed in the fourth column in Supplementary Table 1. The required structure sizes and the corresponding transmittance are also listed below.


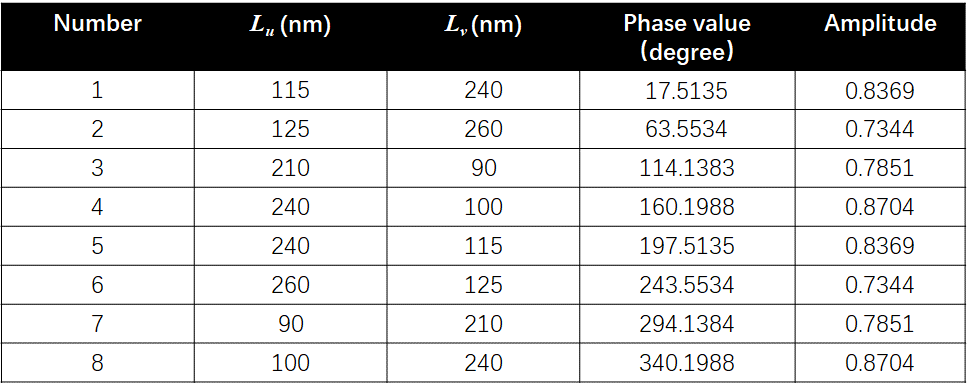


Supplementary Table 1 The parameter table of eight-unit cells. The listed unit cells guarantee the 8-level phase of propagation phase covering 0-2π (0-360 degrees).

**Supplementary Note 3: DNN Structure**

To recover hidden information (*i.e.,* human faces from CelebA) from the superposition of speckle pattern and the authorized key (*i.e*., the QR code), a deep neural network, namely DMNet in this study, is designed as shown in Supplementary Fig. 2. The DMNet comprises of three parts: 1) Input 2 (the QR code) is first transformed by the complex fully connected layer (Com-FC1); 2) Input 1 (the speckle pattern) concatenated with the transformed Input 2 is then fed into the a U-net integrated with densely-connected layers; 3) and the output from 2) is then processed by the other complex fully connected layer (Com-FC2), whose result is the final output of this network. Notably, the use of the complex fully connected layers is to mimic the scattering of the optical field^2^, among which the Com-FC1 resembles the forward scattering process and the Com-FC2 does for the inverse scattering process. For the network in 2), it is similar to the DNN in Ref. [3], a U-net based neural network^4^, with densely connected layers^5^ integrated. To speed up the training process with limited computational resources, plain convolution layers, rather than densely connected layers in all the layers as in Ref. [3], are applied for the down-sampling operations with 4-by-4 kernels, 2-by-2 stride, and 1-by-1 zero padding. The dimensions of the feature maps, output by such convolutional layers, will be parametrically reduced to a half of the input dimension. Layers for the up-sampling operations are modified similarly: transposed convolutional layers are set with 4-by-4 kernels, 2-by-2 stride, and 1-by-1 zero padding. The values of both the targets and inputs are normalized between 0 and 1.


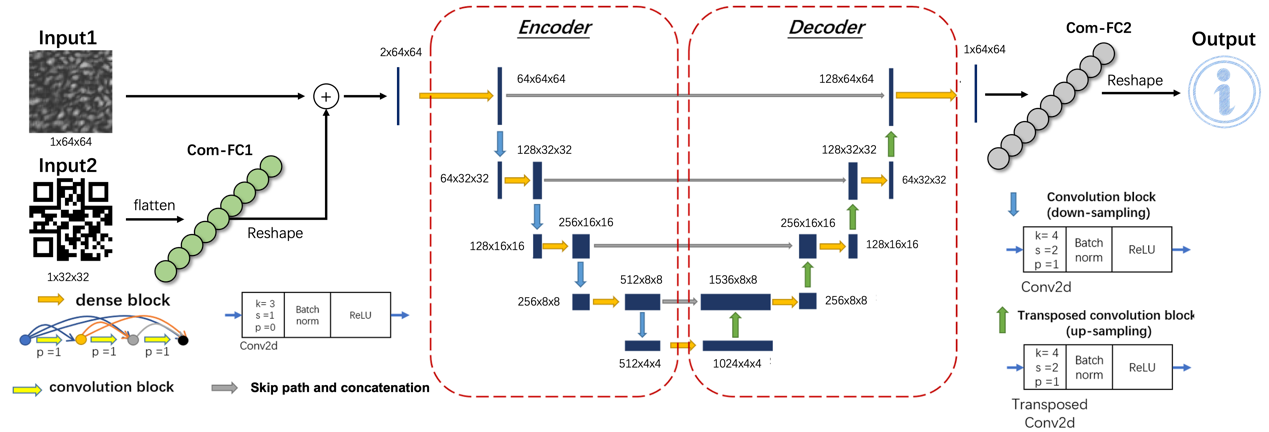


Supplementary Fig. 2 Architecture of the DMNet. It is the combination of 1) a complex-valued fully connected layer (Com-FC1), 2) an U-net integrated with densely connected layers, and 3) a complex-valued fully connected layer (Com-FC2). The network involves two inputs and merely one output: Input1 is the speckle pattern, Input 2 is the QR code, and the output is the decrypted information.

**Supplementary Note 4:** **Quantitative analysis of information complexity for different inputs**

According to the information theory proposed by Shannon, higher the entropy represents more uncertainty in the information or the images, making the information more complicated. The information complexity can be quantified by the information entropy or Shannon entropy, given by

$H\left( I \right)=-\sum_{i} P\left( i \right)\log_{2} P(i)$ (3)

where I is the information (image in this study), P(i) is the probability distribution of grayscale in the image. As calculated, the average entropy for dataset CelebA, fMNIST, quickdraw and MNIST are 7.57, 3.80, 0.825, and 0.692 bits, respectively, with typical examples provided in Supplementary Fig. 3. As seen, the entropy of human face images is larger than that of clothes, symbol, and digits, with much more profound textured and intensity (or grayscale) distribution. Therefore, it is safe to claim that human face images are more complicated information than handwritten digits/texts.


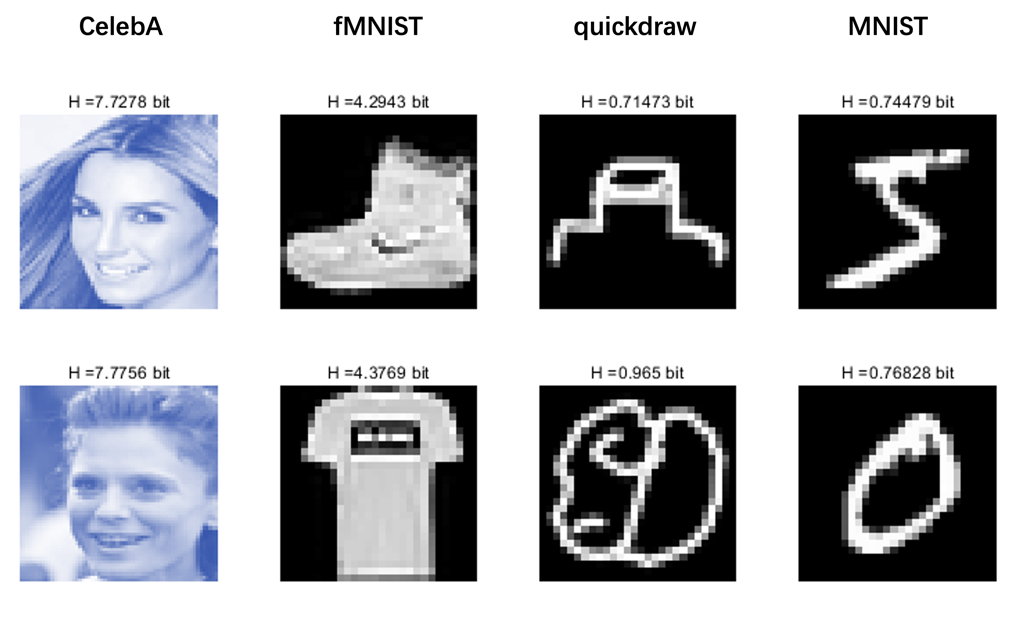


Supplementary Fig. 3 Information entropy of some examples from four different datasets: CelebA, fMNIST, quickdraw and MNIST. The facial images used in this figure were attained using the CelebA database.

**Supplementary Note 5: Decryption performance of DMNet for different datasets**

As complimentary support for the main text, this section provides additional demonstrations regarding two different datasets as plaintext: fMNIST (Supplementary Fig. 4) and Quickdraw (Supplementary Fig. 5). Plaintexts in these two datasets are much simpler than the human faces from CelebA dataset and their decryption performance behaves quite well since almost all the detailed features can be found in the decrypted images. These results confirm that our proposed DMNet can be effectively generalized to different categories of plaintext.


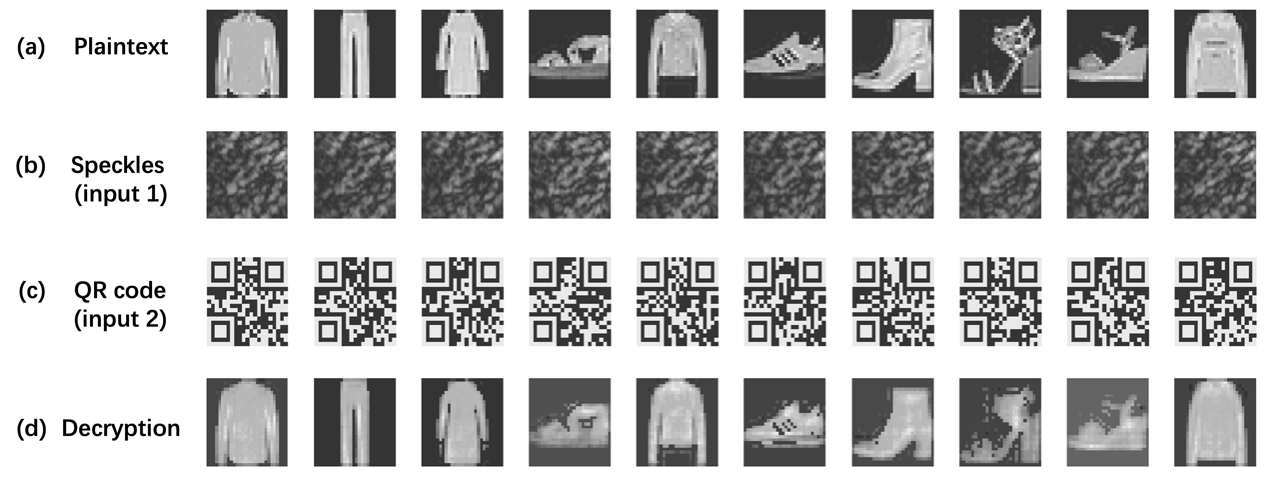


Supplementary Fig. 4 Decryption performance based on DMNet with fMNIST dataset. (a) Plaintext for encryption; (b) The corresponding ciphertext, i.e., the speckles; (c) QR code; (d) The decrypted information by inputting (b) and (c) into the corresponding DMNet. QR: quick response.


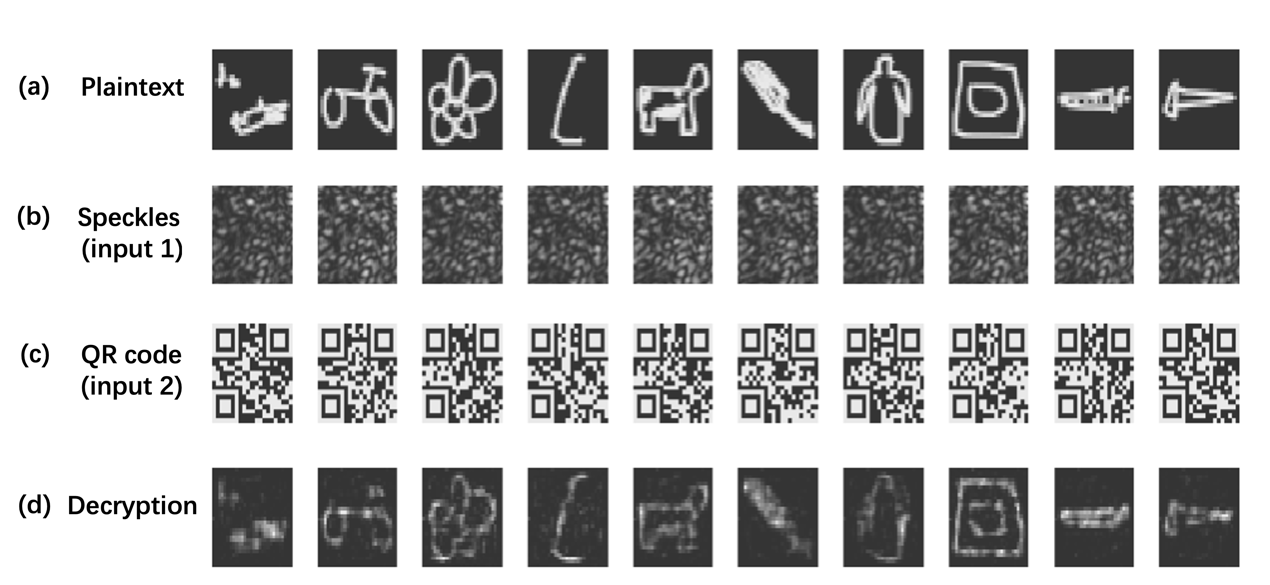


Supplementary Fig. 5 Decryption performance based on DMNet with Quickdraw dataset. (a) Plaintext for encryption; (b) The corresponding ciphertext, i.e., the speckles; (c) QR code; (d) The decrypted information by inputting (b) and (c) into the corresponding DMNet. QR: quick response.

**Supplementary Note 6: Results with mismatched pairs of inputs**

When the input speckle (Input 1) and QR code (Input 2) are matched, high-fidelity outputs can be obtained (the images on the second row). But if Input 1 is kept unchanged while the QR code (denoted as “Mismatched Input 2”) are swapped with the one corresponding to other samples, the network output (denoted as “Mismatched output”) fails to recover the human faces, yet with similar patterns as shown in the main text (Fig. 4a).


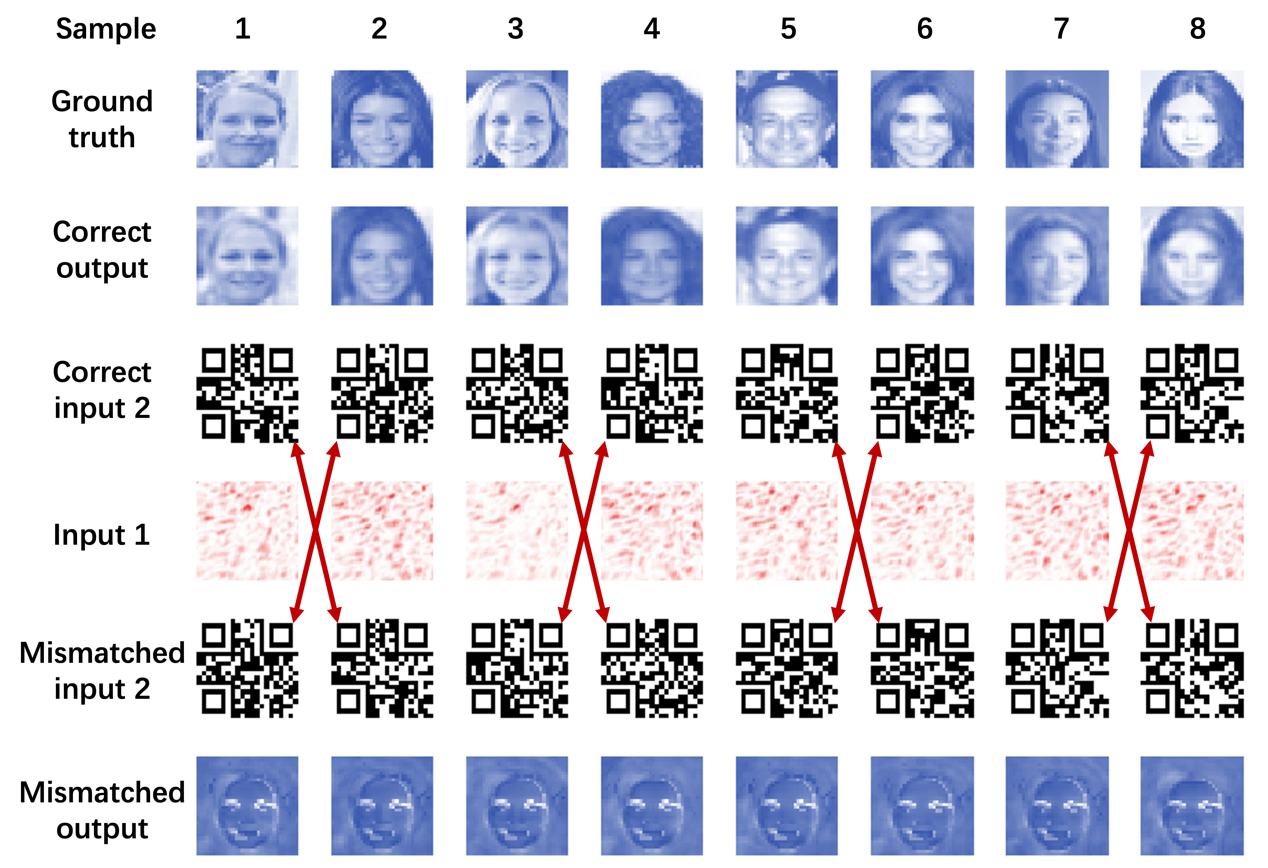


Supplementary Fig. 6 Decryption results with matched and unmatched input pairs. For the unmatched set, Input 1 (speckle pattern) is kept correct but the QR code is switched with its counterpart corresponding to other samples. The facial images used in this figure were attained using the CelebA database.

**Supplementary Note 7: Comparison of speckles and learning efficiency with different types of scattering media**

The output fields from ground glass and DM are both observed as alternately dark and bright grains, which can be referred to Supplementary Fig. 7a and b. The average PCCs of recovered images from DM and ground glass by deep learning with the same epochs (500 epochs) are 0.94 and 0.941, respectively, which cannot support the statement of the reviewer. Further, we also attached the training curves for these two media in Supplementary Fig. 7c, where one cannot see obvious difference between these two curves. In brief, in this deep learning module, there is no clear relationship between the memory-effect range and recovery efficiency.


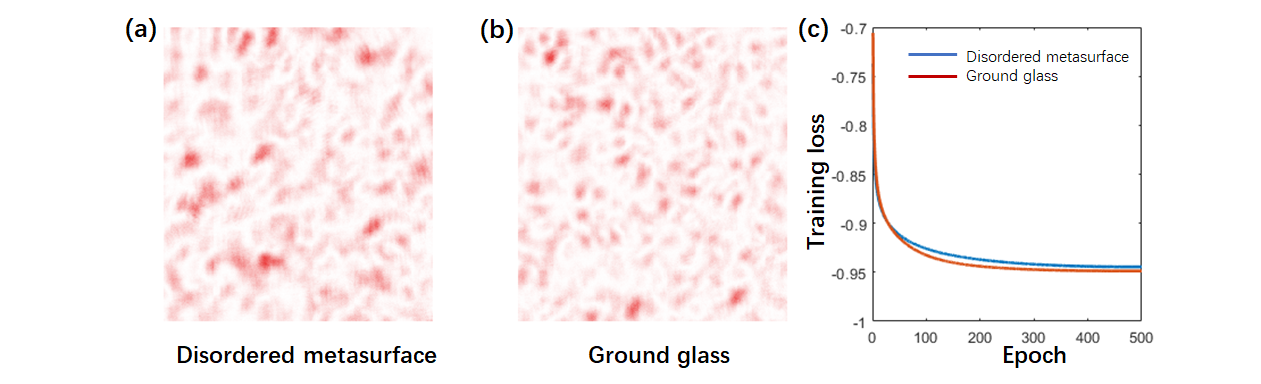


Supplementary Fig. 7 Typical output speckles from a disordered metasurface (DM) (a) and a ground glass diffuser (b). (c) Training loss versus training epoch for data from the DM and the ground glass.

**Supplementary Note 8: Wavelength-sensitive meta-pillars**

The propagation phase of meta-pillar with height of 600 nm is sensitive to the wavelength of light, which can be referred to Supplementary Fig. 8a. In order to generate wavelength-sensitive speckles, meta-pillars with diverse correspondence between the propagation phase and wavelength are screened out. Such a procedure allows the wavelength to serve as another encryption parameter, in which more channels in another dimension can support independent information transmission and thus more DMNets can be trained for these data. To further improve the sensitivity, meta-pillars with greater heights (e.g., 1000 nm) can be considered, as simulated in Supplementary Fig. 8b. As shown, the curve of pillar with height of 1000 nm has a more complex correspondence between the propagation phase and the wavelength compared with its peer with a height of 600 nm. This suggests a more wavelength-sensitive feature.


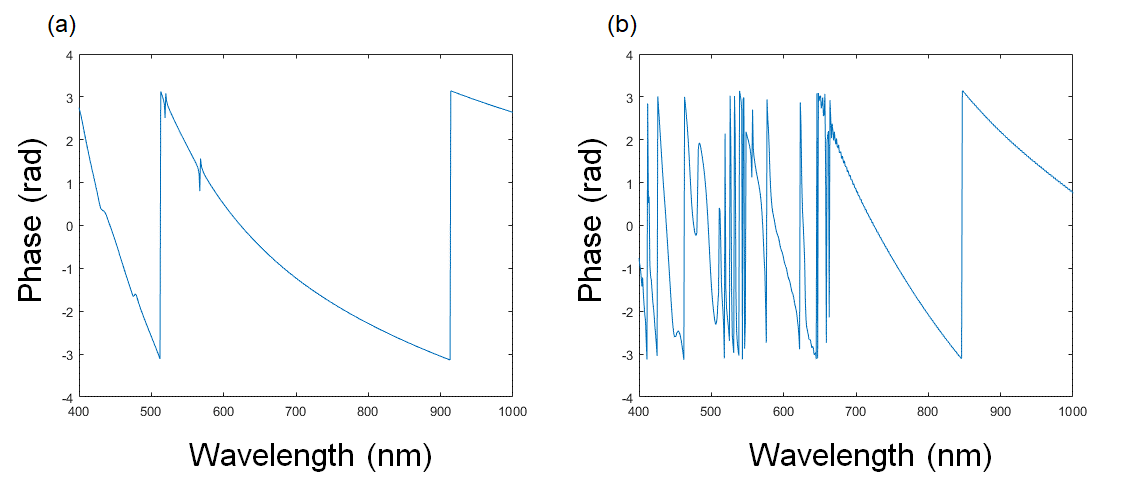


Supplementary Fig. 8 The simulated curves of propagation phase vs. wavelength for meta-pillars with heights of 600nm (a) and 1000nm (b). Periodic constant (P) of 350 nm, and the lengths of two axis (u and v) of meta-pillars are 260 nm and 125 nm.

**Supplementary Note 9: Statistic analysis of output speckles**

Classical transmission matrix modal in optical wavefront shaping can be used to analyze the correspondence between the input and the output speckle in the system. For an optical system with optical scattering, a transmission matrix (*T*) can be used to bridge the relationship between the input ($E_{in}$) and output($E_{out}$)

$E_{out}=T\times E_{in}$ (4)

For a scattering medium with an infinite boundary, the dimension of *T* is usually infinite. In real scenarios, the dimension of the measured transmission matrix (M×N) is determined by the dimensions of the input (M×1) and the output (N×1). The occasion that a constant output profile of waves can be obtained with different incident wave profiles normally happens when M is larger than N. Let us consider an extreme condition: M>>N, N=1, and M is a constant. The output is detected using a single pixel detector and is the interference superposition of all input pixels. In this case, same output can be easily obtained with different input phase profiles using some iterative algorithms like genetic algorithm. With the increase of the number of pixels in the output, such a condition is harder to be met as the rank of T becomes larger. In this experiment, M=1024 and N=65536. We have also provided the statistical histogram about the similarity of speckles from 60,000 human face phase images as below, in which the probability density for similarity to be larger than 0.9 approaches zero.


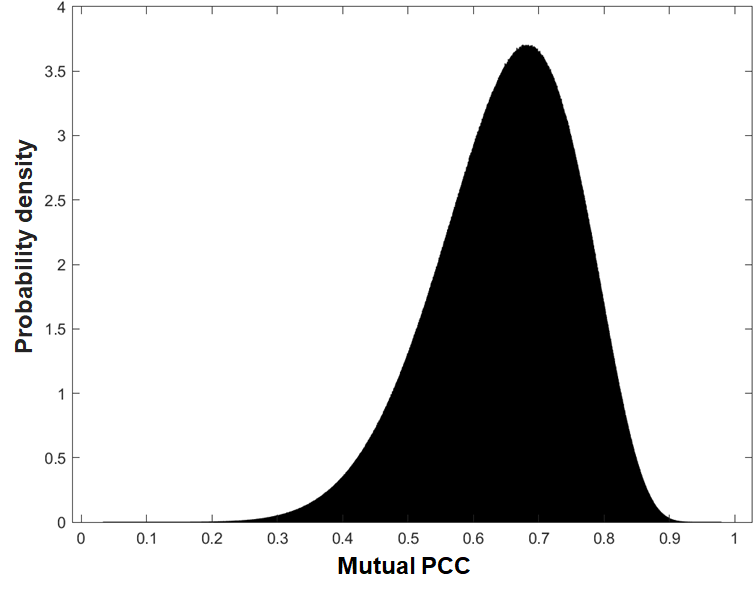


Supplementary Fig. 9 Probability density function of mutual PCC among speckles corresponding to 60,000 human face images. The mutual PCC is calculated based on each pair of speckle patterns generated from different face images.

**REFERENCES**

1. Kang M, Feng T, Wang H-T, Li J. Wave front engineering from an array of thin aperture antennas. *Opt. Exp.* **20**, 15882-15890 (2012).

2. Caramazza P, Moran O, Murray-Smith R, Faccio D. Transmission of natural scene images through a multimode fibre. *Nat. Commun.* **10**, 2029 (2019).

3. Li S, Deng M, Lee J, Sinha A, Barbastathis G. Imaging through glass diffusers using densely connected convolutional networks. *Optica* **5**, 803-813 (2018).

4. Olaf Ronneberger PF, and Thomas Brox. U-Net: Convolutional Networks for Biomedical Image Segmentation. *arXiv:1505.04597*, (2015).

5. Gao Huang ZL, Laurens van der Maaten and Kilian Q. Weinberger. Densely Connected Convolutional Networks. *arXiv:1608.06993*, (2016).
